# Making Sense of Monkeypox: A Comparison of Other Poxviruses to the Monkeypox

**DOI:** 10.7759/cureus.38083

**Published:** 2023-04-24

**Authors:** Harsha Pattnaik, Salim Surani, Lokesh Goyal, Rahul Kashyap

**Affiliations:** 1 Medicine, Lady Hardinge Medical College, University of Delhi, New Delhi, IND; 2 Anesthesiology, Mayo Clinic, Rochester, USA; 3 Medicine, Texas A&M University, College Station, USA; 4 Medicine, University of North Texas, Dallas, USA; 5 Internal Medicine, Pulmonary Associates, Corpus Christi, USA; 6 Clinical Medicine, University of Houston, Houston, USA; 7 Hospital Medicine, Christus Spohn Hospital, Corpus Christi, USA; 8 Global Clinical Scholars Research Training (GCSRT), Harvard Medical School, Boston, USA; 9 Research, Global Remote Research Program, St. Paul, USA; 10 Critical Care Medicine, Mayo Clinic, Rochester, USA; 11 Research, WellSpan Health, York, USA

**Keywords:** pox, orthopoxvirus, pandemic, outbreak, zoonoses, smallpox, monkeypox

## Abstract

The current monkeypox (MPX) outbreak has been declared a public health emergency of international concern (PHEIC) by the World Health Organization (WHO). It is a zoonotic disease that has persisted in the African basin for decades but suddenly exploded into the international sphere this year. In this paper, we provide a comprehensive overview of monkeypox, including a hypothesis of the rapid spread of the virus, its epidemiology and clinical features, a comparison with other orthopoxviruses such as chickenpox and smallpox, past and present outbreaks, and strategies for its prevention and treatment.

## Introduction and background

Monkeypox (MPX) is a zoonotic virus belonging to genus *Orthopoxvirus*, with occasional human outbreaks occurring sporadically over the years and, most importantly, in the current time. Monkeypox (MPX) has mostly been confined to Central and West Africa, which has led to it being neglected despite causing multiple outbreaks in recent times. With globalization and rapid connectivity across the world, the transcontinental spread of MPX has brought it to the global forefront with numbers still on the rise. Here, we provide a brief overview of monkeypox and the lessons that can be learned from past outbreaks to deal with the current situation.

In 1958, MPX was first isolated and identified in captive monkeys in a research facility, hence the name [1]. But it can be found in a variety of mammals including but not limited to primates, prairie dogs, squirrels, rats, mice, and humans [2-6]. Wild rodents in the rainforests of Africa appear to be the natural reservoirs of the virus, and outbreaks can be linked to them [7,8].

It causes a disease similar to other poxviruses and is characterized by a pustular rash and lymphadenopathy. At a time when the smallpox epidemic was in its dying stages, the first human case of MPX was identified. In 1970, a nine-month-old child in the Democratic Republic of the Congo (DRC), initially thought to be a case of smallpox, was found to harbor the MPX virus [9]. Since then, MPX has become endemic to the DRC with maximum cases reported from there.

## Review

Epidemiology and transmission

In 1984, the World Health Organization (WHO) considered MPX a rare sporadic zoonotic disease with limited capacity to spread between humans [10]. However, over the course of years, it has caused multiple outbreaks in Central and West Africa and in a viral crescendo has now spread worldwide.

A Congolese study in 2014 analyzed the diversity of the MPX viral genome with samples from primary and secondary human cases and identified four distinct lineages within the Central African clade. They discovered in 17% of the samples that through genomic destabilization and gene loss, there was increased disease transmissibility and severity with the potential for accelerated spread through humans [11]. The warning signs for a widespread MPX outbreak have been here all along, and we must take lessons from African countries that have been dealing with this disease since the early 1980s.

The MPX virus has two distinct genetic clades; West African and Central African (a.k.a. Congo Basin clade), which have differences not only in epidemiology but also in symptomatology. Reports suggest that the Central African clade is more virulent, causing more severe forms of disease. A systematic review of human MPX epidemiology reports that the Central African clade has a case fatality ratio (CFR) of up to 10.6% and human-to-human transmission of up to six sequential events. Comparatively, the West African clade is milder with a significantly lower CFR of 3.6% [12]. The ongoing global MPX outbreak is of the West African clade, which accounts for milder disease in the affected.

Multiple outbreaks have been seen in the Central African Republic [13-15], the Democratic Republic of the Congo [16,17], the Republic of the Congo [18], South Sudan [19], and Nigeria [20,21], wherein both the clades have shown significant human-to-human transmission. The mathematical modelling of human-to-human transmission found that monkeypox had epidemic potential, with a reproduction number (R0) of >1 [22].

The transmission of the MPX virus can occur through multiple routes. Direct contact with skin lesion exudate or crust, fomites, bodily fluids, and respiratory droplets can lead to the spread of the virus. Viral shedding can occur via fecal matter [23,24]. Sexual contact can also lead to spread, and a disproportionately higher number of males who have sex with males (MSM) have been affected in the current outbreak [25]. Often, young males and males who hunt for prey and come into contact with infected animals are the ones predominantly affected in African communities.

Population explosion and deforestation, which led to more contact between wild rodents carrying the MPX virus and humans, as well as the waning immunity in the community against orthopoxviruses in the post-smallpox vaccination era appear to be the main reasons behind the upsurge of MPX cases.

It was mainly considered to be a disease of the young. But the median age at presentation has increased from four (1970s) to 21 years (2010-2019), an interesting relationship with the cessation of smallpox vaccination, which has been explored herein [12].

The smallpox-monkeypox tandem

With the cessation of smallpox immunization, there has been an increasing trend of other orthopoxvirus infections. With the decrease in the herd immunity against poxviruses, outbreaks of related viruses such as monkeypox were inevitable. Vaccinia virus vaccine for smallpox kept other poxvirus outbreaks in check by offering cross-immunity. With the eradication of smallpox by 1980 and the decline in smallpox virus vaccination drive, the outbreaks of monkeypox viruses have seen an upswing due to declining immunity [26].

Interestingly, in the early era of MPX in 1970-1990, it was mainly a disease of children with a median age at presentation of 4-5 years. In the 2000s, this increased to 10 years of age and then to 21 years in 2010-2019. In the 1980s, 100% of deaths were in children younger than 10 years of age, while this number dropped to 37.5% of total deaths in 2000-2019 indicating increasing mortality and susceptibility in older individuals than before [27]. These data trends closely follow the global intensified smallpox eradication program that began in 1967 and the cessation of routine smallpox vaccination in the 1980s following its eradication [28,29].

The ones affected in the early years of MPX, mainly children 4-5 years, were affected as they were infected before being vaccinated. The age increased to 10 years in the 2000s as those mainly affected were individuals who missed the time frame of smallpox vaccination, and adults older than 20 years would have had a history of vaccination, leaving those younger vulnerable. Now, about 40 years since the cessation of smallpox immunization, even adults are affected in the more recent outbreaks, and this resurgence of monkeypox can in part be contributed to the cessation of smallpox immunization alongside increased overpopulation and deforestation, which increased the contact with wild rodents carrying the virus.

Additionally, smallpox dominated the 1970s-1980s, which could have led to the masking of monkeypox cases due to similar presentations. Irrespective of such speculations, both the prevalence and incidence of MPX have increased since the discontinuation of routine smallpox vaccination, though precise data are hard to obtain due to the shortcomings in disease reporting and confirmation [2,30]. Mathematical modelling, in the context of decreasing herd immunity against orthopoxviruses in the post-smallpox vaccination era, reflects an increasing threat of interhuman transmission and community spread [22].

MPX vaccination

It is presumed that smallpox vaccination provides up to 85% cross-protection against monkeypox, though the duration of immunity it provides is unknown. The United States currently has two vaccines in its arsenal to prevent smallpox: ACAM2000 (single-dose live replicating vaccinia virus) and JYNNEOS (double-dose live non-replicating modified vaccinia virus).

ACAM2000 carries a high risk of causing viremia itself. Hence, the Ankara-JYNNEOS vaccine given as two doses, four weeks apart, has a superior safety profile compared to other vaccines as it does not create a skin lesion or carry a risk for local or disseminated spread [31]. Additionally, clinical trials show better safety and immunizing capacity in immunocompromised patients against monkeypox [32].

At present, vaccination is recommended for those at risk of exposure to orthopoxviruses, and these vaccines surely can play a role in the ongoing MPX outbreak, though they are yet to be recommended for routine preventive use in first-line personnel or community for MPX. Close contacts of MPX-infected individuals can be given postexposure vaccination. The Centers for Disease Control and Prevention (CDC) recommends vaccination within four days of exposure to prevent disease or up to 14 days after exposure to reduce the severity of disease [33].

MPX prevention

Due to the risk of droplet- and fomite-based transmission of infection, the CDC recommends the isolation of patients in negative pressure room and standard contact and droplet precautions. Avoiding direct contact with skin lesion and personal use materials contaminated with fomites can prevent infection. The use of personal protective equipment such as gowns, gloves, masks, and eye protection is of paramount importance in those directly handling patients [34].

Clinical picture

Once the virus enters the human body, either through the mucus membranes or through direct skin contact, it replicates at the inoculation site and then spreads to the lymph nodes. From here, the virus spreads and seeds into other organs. This is the incubation period, which typically lasts anywhere from seven to 14 days with a maximum period of 21 days.

The prodromal phase consists of symptoms such as fever, chills, headache, myalgia, fatigue, sore throat, and lymphadenopathy. Lymphadenopathy, which often presents early alongside fever, is the one distinct characteristic that differentiates MPX from variola and other poxviruses.

A rash appears 1-3 days after fever onset, mostly in the peripheral parts of the body, but can be disseminated throughout the body in severe case. Serum antibodies are often detectable by the time lesions appear [35]. The disease lasts up to 2-4 weeks until the lesions desquamate. After the crusts fall off, the patient is no longer contagious. In most cases, the disease is mild and self-limiting [36,37].

Rash Evolution

After 1-3 days, after the onset of fever and lymphadenopathy, the oropharyngeal mucosa is the first site to be affected, followed by eruptions in the skin. The rash is mainly peripheral, involving the face and extremities including the palms and soles, displaying a centrifugal pattern, and may spread to involve the rest of the body in few cases. Lesions appear in crops and change synchronously. The rash is initially maculopapular, which evolves into vesico-pustular type. Typical lesions are characterized as firm, deep-seated, and about 2-10 mm in size. Lesions remain in the pustular phase for 5-7 days. These lesions then ulcerate and crust before healing over several weeks with or without scarring. The number and density of lesions vary and can range from a few to thousands [31].

Individuals vaccinated with smallpox vaccine who subsequently contracted monkeypox have been noted to have fewer and smaller lesions with less spread of lesions across the body and less post-lesion sequelae [38].

In the current outbreak, initial lesions are seen on or near the groin and anus, which suggests sexual contact as a mode of viral inoculation. Anogenital lesions are usually maculopapular or vesicular and painful until they start crusting. They might be associated with inguinal lymphadenitis.

Diagnostic Criteria

Table 1 shows the CDC’s definitions for the 2022 monkeypox outbreak.

**Table 1:** The Centers for Disease Control and Prevention (CDC) definitions for the 2022 monkeypox outbreak *The characteristic rash associated with monkeypox lesions involves the following: deep-seated and well-circumscribed lesions, often with central umbilication, and lesion progression through specific sequential stages: macules, papules, vesicles, pustules, and scabs; this can sometimes be confused with other diseases that are more commonly encountered in clinical practice (e.g., secondary syphilis, herpes, and varicella zoster). Historically, sporadic accounts of patients co-infected with the monkeypox virus and other infectious agents (e.g., varicella zoster and syphilis) have been reported, so patients with a characteristic rash should be considered for testing, even if other tests are positive ^Within 21 days of illness onset: reports having contact with a person or people with a similar appearing rash or who received a diagnosis of confirmed or probable monkeypox OR had close or intimate in-person contact with individuals in a social network experiencing monkeypox activity, which includes males who have sex with males (MSM) who meet partners through an online website, digital application (“app”), or social event (e.g., a bar or party), OR traveled outside the United States to a country with confirmed cases of monkeypox or where the monkeypox virus is endemic OR had contact with a dead or live wild animal or exotic pet that is an African endemic species or used a product derived from such animals (e.g., game meat, creams, lotions, and powders). Exclusion criteria: a case may be excluded as a suspect, probable, or confirmed case if an alternative diagnosis can fully explain the illness OR an individual with symptoms consistent with monkeypox does not develop a rash within five days of illness onset OR a case where high-quality specimens do not demonstrate the presence of orthopoxvirus or monkeypox virus or antibodies to orthopoxvirus †Presentation is consistent with illnesses confused with monkeypox (secondary syphilis, herpes, and varicella zoster)

Type of case	Definition
Suspect case	New characteristic rash* OR meets one of the epidemiological criteria^ and has a high clinical suspicion† for monkeypox
Probable case	No suspicion of other recent orthopoxvirus exposure (e.g., vaccinia virus in ACAM2000 vaccination) AND the demonstration of the presence of orthopoxvirus DNA by the polymerase chain reaction of a clinical specimen OR orthopoxvirus using immunohistochemical or electron microscopy testing methods OR the demonstration of detectable levels of anti-orthopoxvirus immunoglobulin M (IgM) antibody during the period of 4-56 days after rash onset
Confirmed case	The demonstration of the presence of monkeypox virus DNA by polymerase chain reaction testing or next-generation sequencing of a clinical specimen OR the isolation of monkeypox virus in culture from a clinical specimen

Clinical suspicion for monkeypox should arise if there is presence of a characteristic rash and it fulfills the epidemiological criteria. The monkeypox rash comprises deep-seated and well-circumscribed lesions with central umbilication. There are sequential stages of rash evolution: macules, papules, vesicles, pustules, and scabs. The diagnosis of monkeypox is strengthened if, within 21 days of illness onset, there is history of having contact with a person or people with a similar appearing rash or who received a diagnosis of confirmed or probable monkeypox or had close or intimate in-person contact with individuals in a social network experiencing monkeypox activity (e.g., males who have sex with males) or have history of travel to a country with confirmed cases of monkeypox or where the virus is endemic or had contact with an animal of African origin.

The diagnosis of monkeypox can be excluded if an alternative diagnosis appears more probable like other orthopox infections or an individual with prodromal symptoms does not go on to develop a rash within five days of illness onset or the microbiological test of specimens does not demonstrate an evidence of monkeypox infection.

There have been various reports of monkeypox co-infection with herpes simplex [39], syphilis [40,41], and chickenpox [42]. Hence, the diagnosis of monkeypox should not be easily dismissed in case of other more common ailments if a characteristic rash is seen.

Complications

Most cases of monkeypox are self-limiting and resolve without many complications except occasional scarring and hyper/hypopigmentation. There could be bacterial superinfection of the lesions; corneal involvement can lead to permanent scarring and vision loss. Children younger than eight years, pregnant females, and immunocompromised individuals may suffer from multisystem involvement leading to pneumonia, encephalitis, gastrointestinal involvement leading to sepsis, dehydration (due to diarrhea, vomiting, and fluid loss from the loss of skin barrier), and even death. Studies have identified the mortality rate being close to 10%, and mainly children were affected [16,17,19,21,30,43].

Differentials

Any rash-causing illness can be a differential diagnosis for monkeypox. Chickenpox is the most common misdiagnosis in monkeypox cases (up to 50% of suspected MPX cases in the DRC [44,45]). Co-infections with both monkeypox and chickenpox have been extensively reported [44,46-48] and are quite common in populations not vaccinated for varicella.

Another strong contender for monkeypox differential is smallpox. Smallpox and monkeypox (Table 2) have almost identical features and can only be distinguished by the presence of lymphadenopathy in MPX, which is absent in smallpox. This similar clinical picture in the context of uprise in monkeypox outbreaks can lead to masquerading in the case of a potential smallpox outbreak in terms of laboratory accident of variola reserves or an act of bioterrorism.

**Table 2:** Comparison of smallpox, chickenpox, and monkeypox

Features	Monkeypox	Smallpox	Chickenpox
Causative agent	Monkeypox virus (orthopoxvirus)	Variola virus (orthopoxvirus)	Varicella virus (herpesvirus)
Incubation period	5-21 days	7-19 days	10-21 days
Prodromal period	Yes, 1-3 days	Yes, 2-4 days	Maybe, 0-2 days
Fever	Yes, between 38.5°C and 40.5°C	Yes, high grade, >40°C	Yes, low grade, <38.8°C
Lymphadenopathy	Yes	No	No
Oropharyngeal involvement	Lesions begin in the oropharynx	Lesions begin in the oropharynx	No lesions in the oropharynx
Rash onset	Rash appears 1-3 days after fever onset	Rash appears 2-4 days after fever onset	Rash can appear without prodrome phase
Rash characteristics	Hard, deep-seated, and well-circumscribed lesions, centrally umbilicated	Hard, deep-seated, and well-circumscribed lesions, centrally umbilicated	Soft, superficial, and easily ruptured
Rash progression	Lesions progress through specific sequential stages: macules, papules, vesicles, pustules, and scabs; lesions are often in the same stage throughout the body with each stage lasting 1-2 days	Lesions progress through specific sequential stages: macules, papules, vesicles, pustules, and scabs; lesions are often in the same stage throughout the body with each stage lasting 1-2 days	Lesions are often in different stages of progression synchronously on the body and progress to subsequent stages rapidly
Rash distribution	Centrifugal, the face and extremities involved initially	Centrifugal, the face and extremities involved initially	Centripetal, lesions spread outward from the trunk to the extremities and face
Lesions on the palms or soles	Yes	Yes	Rare
Duration of illness	Up to 2-4 weeks after rash onset	Up to four weeks after rash onset	Up to two weeks after rash onset
Risk of scarring	Low risk, hypo/hyperpigmentation and keloids	High risk, deep pitted scars and potentially disfiguring	Low risk, minor pock marks left behind
Mortality	Low, ranging from 1% to 10%	High, close to 30%	Very low, 0.03 per million population

Besides the above two, MPX can be misdiagnosed as disseminated herpes simplex, zoster or vaccinia, syphilis, yaws, measles, rubella, rickettsialpox, cutaneous anthrax, fungal infection in HIV patients, bacterial skin infections such as staphylococcus, and drug-associated eruption.

Current diagnostics

Patients presenting with symptoms suggestive of monkeypox should undergo confirmatory diagnostic testing. Nucleic acid amplification tests (NAAT), specifically real-time polymerase chain reaction (RT-PCR), detect targeted sequences in the viral genome and identify monkeypox infections and reliably differentiate it from other poxvirus infections. PCR can be used alone or in combination with genomic sequencing [49-51]. Swab samples from skin lesions, the roof, exudates, or fluid of vesicles and pustules or crusts or scabs, are considered optimal for the detection of monkeypox virus and correlate with the infectivity and clinical course of infection [52-54]. Although not necessary, skin biopsy is also an option. Due to the very short duration of viremia, blood tests are often unreliable in detecting the evidence of monkeypox and are hence not recommended. Additionally, antibody detection methods cannot be used to detect MPX since orthopoxviruses are serologically cross-reactive and hence would lead to nonspecific findings and potentially be false positive in patients who had previous chickenpox infections or were vaccinated for smallpox in the past. Electron microscopy is another uncommon modality and could be used to distinguish monkeypox, which is an orthopoxvirus from its most common differential diagnosis, chickenpox, which is a herpesvirus [55].

Treatment of monkeypox

Even though human MPX cases appeared in the 1970s, no standard clinical management guidelines or specific drugs have been created. Like most viral illnesses, the treatment is mostly supportive symptom management [56].

In severe cases, pregnant females, children younger than eight years, immunocompromised patients, and those with smallpox vaccination contraindications, experimental therapeutics with benefit against orthopoxviruses can be tried. Brincidofovir and cidofovir, DNA polymerase inhibitors, tecovirimat, intracellular virus release inhibitor, and intravenous vaccinia immune globulin have debatable efficacy against MPX [31,57].

As per the CDC’s expanded access investigational new drug (EA-IND) protocol, two antiviral agents, brincidofovir and tecovirimat, which had been approved by the FDA for the treatment of smallpox in 2021 and 2018, respectively, can be considered for the treatment of related orthopoxvirus infections including monkeypox.

The efficacy of these antiviral agents and intravenous vaccinia immune globulin in the management of monkeypox remains to be seen, and their use must be carefully evaluated against their side effect profile [57].

Outbreaks: Past and present

1970-1990

After the identification of the first human MPX case, a few cases (ranging from one to 10) have been reported in the countries of West and Central Africa, such as the Central African Republic [58], Nigeria [59-61], Cameroon [62], Liberia [61], Gabon [63,64], and Sierra Leone [61]. But the majority of burden of cases (>386 confirmed) [62] had come from the Democratic Republic of the Congo (DRC, formerly Zaire) where the disease was first discovered and has been endemic ever since.

1991-2000

Due to the lack of reporting, the cases in the 1990s could be underestimated. Five hundred eleven [63,65] cases were reported from the DRC with other countries that previously not reported any cases.

2000s

The first major outbreak of MPX outside of Africa happened in the Midwest in the United States in 2003. Prairie dogs were housed with infected Gambian pouched rats imported from Ghana and subsequently spread it to humans they came in contact with. About 71 humans were affected, and the manifestations were mostly mild with no deaths recorded, which is attributed to the fact that the strain was of West African clade. No human-to-human transmission was found during this outbreak, and all cases were due to direct contact with the infected dogs [66,67].

Contrary to the MPX outbreaks in Africa, where children were disproportionately affected, in the US outbreak, mostly adults were affected. This is interesting, especially when it was found that age and smallpox vaccination had little influence on the disease manifestations contrary to studies conducted in African countries. Nearly one-third of infected adults had a history of smallpox immunization [68]. Further, it was reported that the manner of exposure (invasive or noninvasive) influenced disease severity [69].

In this outbreak, the CDC had authorized the emergency use of smallpox vaccine, cidofovir, and vaccinia immunoglobulin [66].

The first reported outbreak was from South Sudan with 49 cases affected by MPX [70]. Cases continued to rise in the DRC, and due to the lack of proper reporting and surveillance, the cases are severely underestimated and not fully enumerable [30,44,47].

2010-2020

The first large outbreak outside of the DRC happened in Nigeria between 2017 and 2018 with 244 cases reported out of which 101 were confirmed to be MPX [71].

In September 2017, cases began to rise in Nigeria in what is considered to be the largest recorded outbreak caused by the West African clade. Ever since then, hundreds of cases have been reported. Interestingly, many cases appear to be males who have genital lesions, which suggests human-to-human transmission via sexual route as opposed to direct contact, which was purported to be the major mode of infection [72].

In 2018, the disease was imported from Nigeria to the United Kingdom by two infected travelers who subsequently caused a documented secondary MPX infection in a healthcare worker [73]. One case was reported in Israel in October 2018 [74] and another in Singapore in May 2019 [75], which were both linked to males who had traveled from Nigeria. Cases continued to multiply in the DRC [71], and the Central African Republic reported an outbreak of at least 68 cases [71] as did the Republic of the Congo with 98 probable cases [76].

2021-2022: Leading Up to the Current Outbreak of May 2022

In May 2021, a family traveled from Nigeria to the United Kingdom, and three members brought the infection back with them. Interestingly, they developed symptoms in a sequential manner (day 0, day 19, and day 33), which suggests human-to-human transmission [77]. A case was reported in Texas in July [78] and another in Maryland in November [79], which were both linked to males who had traveled from Nigeria.

Current outbreak

**Figure 1:**
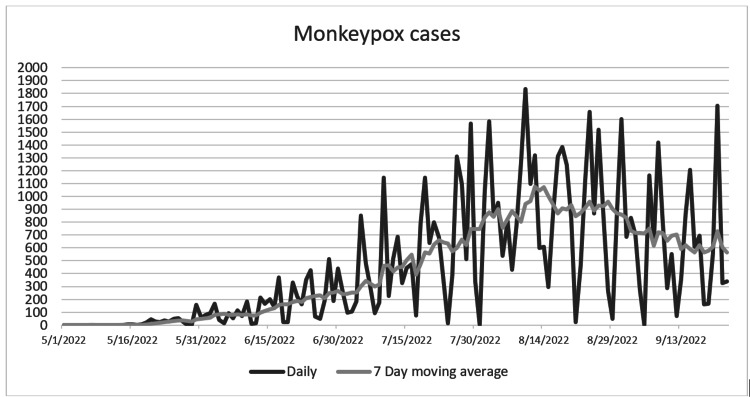
Daily and seven-day rolling average global trends of monkeypox cases [85]

In May 2022, the World Health Organization (WHO) declared an ongoing monkeypox outbreak in non-endemic countries [80] beginning with a cluster of cases reported in the United Kingdom. The first confirmed case was in a male who had traveled from Nigeria, where the disease is endemic [81]. From mid-May, an increasing number of cases were reported from across the globe where the disease is not endemic, mostly concentrated in Europe and North and South America, West Asia, Africa, and Australia. A total of 2,680 cases had been confirmed as of June 20, 2022 (Figure 1) [82]. On July 23, 2022, the WHO declared monkeypox as a public health emergency of international concern (PHEIC). This is the seventh declaration of a PHEIC since 2005 (Table 3) [83,84].

**Table 3:** Top 10 countries with monkeypox cases [84]

	Cases as of September 23, 2022
World	64,881
United States	24,198
Brazil	7,205
Spain	7,083
France	3,943
Germany	3,590
United Kingdom	3,412
Peru	2,251
Colombia	1,653
Canada	1,388
Mexico	1,367

Although the outbreak is considered to be a mild variant of MPX, 72 deaths have been reported from eight countries in the WHO African Region as of June 8, 2022 [85,86]. The majority of cases in the ongoing outbreak have been in Europe contrary to African countries where the disease is endemic, which could be due to underreporting or community immunity to MPX.

The current outbreak displays a worrying trend of extensive human-to-human transmissibility as the majority of reported cases have no history of contact with infected animals or travel to an endemic country. Interestingly, most cases have been among males who have sex with males, suggesting infection through sexual route or through intimate contact, but the mechanisms need to be explored [25]. In the current outbreak, fewer lesions have been reported compared to endemic cases with more lesion diversity in various stages of evolution, more ulcerated lesions, and an almost exclusively peri-genital and/or perianal distribution [87]. The virus has been successfully detected in semen, and viral DNA was found in saliva, nasopharyngeal secretions, urine, feces, and rectal swab [88].

## Conclusions

The ongoing COVID-19 pandemic and the current monkeypox outbreak paint a worrying picture of zoonotic-origin viruses spreading to humans. There was a report of SARS-CoV-2 and monkeypox co-infection in an HIV-positive individual. The increased contact between humans and wild animals, due to the displacement of wild habitats because of deforestation and globalization, as well as the hunting of animals for food and sport or poaching, has increased the possibility of these viruses reaching humans. The rising human population and better connectivity across the globe also provide a fertile ground for the viruses to transmit and mutate as they spread from host to host, leading to a grim prospect of more outbreaks and pandemics to follow.

There is an urgent need for the active surveillance of any illness clusters being reported so that proper measures could be taken to contain the pathogen and prevent the transcontinental spread that has been seen in recent times. Additionally, further large-scale studies are needed to understand the disease process in human beings and explore the spectrum of clinical presentations to better manage this emerging virus and be prepared for the next outbreak.
